# Mid-infrared photoacoustic brain imaging enabled by cascaded gas-filled hollow-core fiber lasers

**DOI:** 10.1117/1.NPh.11.4.045012

**Published:** 2024-11-26

**Authors:** Cuiling Zhang, Kunyang Sui, Marcello Meneghetti, Jose Enrique Antonio-Lopez, Manoj K. Dasa, Rune W. Berg, Rodrigo Amezcua-Correa, Yazhou Wang, Christos Markos

**Affiliations:** a Technical University of Denmark, DTU Electro, Lyngby, Denmark; b University of Copenhagen, Department of Neuroscience, Copenhagen, Denmark; c University of Central Florida, CREOL, The College of Optics and Photonics, Orlando, Florida, United States; d NKT Photonics A/S, Birkerød, Denmark; e NORBLIS ApS, Virum, Denmark

**Keywords:** photoacoustic microscopy, lipids, myelin, mid-infrared, gas-filled hollow-core fiber laser

## Abstract

**Significance:**

Extending the photoacoustic microscopy (PAM) into the mid-infrared (MIR) molecular fingerprint region constitutes a promising route toward label-free imaging of biological molecular structures. Realizing this objective requires a high-energy nanosecond MIR laser source. However, existing MIR laser technologies are limited to either low pulse energy or free-space structure that is sensitive to environmental conditions. Fiber lasers are promising technologies for PAM for their potential to offer both high pulse energy and robust performance, which however have not yet been used for PAM because it is still at the infant research stage.

**Aim:**

We aim to employ the emerging gas-filled anti-resonant hollow-core fiber (ARHCF) laser technology for MIR-PAM for the purpose of imaging myelin-rich regions in a mouse brain.

**Approach:**

This laser source is developed with a high-pulse-energy nanosecond laser at 3.4μm, targeting the main absorption band of myelin sheaths, the primary chemical component of axons in the central nervous system. The laser mechanism relies on two-order gas-induced vibrational stimulated Raman scattering for non-linear wavelength conversion, starting from a 1060-nm pump laser to 3.4  μm through the two-stage gas-filled ARHCFs.

**Results:**

The developed fiber Raman laser was used for the first time for MIR-PAM of mouse brain regions containing structures rich in myelin. The high peak power of ∼1.38  kW and robust performance of the generated MIR Raman pulse addressed the challenge faced by the commonly used MIR lasers.

**Conclusions:**

We pioneered the potential use of high-energy and nanosecond gas-filled ARHCF laser source to MIR-PAM, with a first attempt to report this kind of fiber laser source for PAM of lipid-rich myelin regions in a mouse brain. We also open up possibilities for expanding into a versatile multiwavelength laser source covering multiple biomarkers and being employed to image other materials such as plastics.

## 1 Introduction

Photoacoustic microscopy (PAM) is a prominent non-invasive imaging modality that uses optical excitation to generate ultrasound signals, allowing the visualization of biomedical tissues, inorganic materials, or complex samples with larger penetration depths when compared with other optical imaging techniques.^1^^–^^4^ In recent years, PAM within the visible and near-infrared (NIR) wavelength regions have unveiled a wealth of functional information, contributing to advancements in various research fields.^5^^–^^9^ Currently, the scientific community focuses on moving PAM technology to the mid-infrared (MIR) wavelength domain, opening the door to new opportunities in microscopy with emphasis on probing specific molecular vibrational bands, such as those associated with the ${\mathrm{CH}}_{2}$ groups.^10^^–^^15^ For instance, He et al.^10^ reported the use of mid-infrared photoacoustic microscopy (MIR-PAM) for mapping the lipid compositions (${\mathrm{CH}}_{2}$ stretching transition) in mouse brain and kidney tissue at ∼3.4  μm. Furthermore, MIR-PAM enabled the imaging of carbohydrates (at ∼9.2  μm), lipids (at ∼3.5  μm), and proteins (at ∼6.4  μm) in living cells and tissues.^11^^,^^12^ Compared with the NIR-PAM, the lateral resolution of the MIR-PAM is diffraction-limited to the long wavelength of the MIR laser. This challenge was recently addressed by adding an ultraviolet (UV)-pulsed laser as a probe to enhance the PAM resolution down to the nanometer scale to image lipids, proteins, and nucleic acids.^13^ Compared with MIR photothermal microscopy (MIR-PTM) with a similar underlying mechanism, MIR-PAM achieves higher sensitivity and higher signal-to-noise ratio (SNR) due to the fact that the photoacoustic signal change has a significantly stronger effect than the photothermally induced refractive index change.^10^^–^^13^ A higher proportion of the initial irradiation energy can be detected in the form of photoacoustic (PA) signals, thus allowing detection from larger depths. Despite the high water attenuation in the MIR wavelength range, MIR-PAM can achieve an imaging penetration depth of up to ∼600  μm, whereas for MIR-PTM, it is typically at only tens of micrometers.^16^^,^^17^ Although MIR-PAM has limited spatial resolution compared with MIR-PTM because the latter employs a visible or NIR laser beam to detect the MIR thermal lensing effect, this issue can be mitigated by combining MIR-PAM with UV laser pulses to achieve a higher resolution.^10^^–^^13^

Quantum-cascaded lasers (QCLs) and optical parametric oscillators (OPOs) are the main light sources that have been used for MIR-PAM because mainly of their broad wavelength selectivity.^10^^–^^15^ However, QCL technology is limited by a low peak power of only few watts, leading to a low pulse energy of only few nanojoules at even tens of nanosecond pulse duration.^11^^,^^14^^,^^15^ Some efforts have been made to circumvent this issue, including mitigating the laser energy attenuation caused by the ambient gas absorption such as creating a nitrogen ($N_{2}$)-filled atmosphere and prolonging the pulse duration to tens of nanoseconds. Based on these methods, QCL has been used for scanning mode–based PAM; however, the pulse energy is too low to be used for real-time PA modalities.^14^^,^^15^^,^^18^ OPO technology, on the other hand, can deliver high pulse energy of tens of microjoules. However, its free-space structure^10^ introduces practical limitations due to its sensitivity to environmental conditions such as vibrations, humidity, and temperature.

Fiber lasers are promising alternatives for PA technology because they have the potential to deliver high pulse energy with robust performance against environmental conditions, thanks to their good heat dissipation capability, compact structure, and small weight. Driven by these advantages, mature visible and NIR fiber laser technologies have been recently proposed for PA technology.^19^^–^^22^ However, MIR high-energy fiber lasers have not yet been used for PAM. The limitations of the current MIR fiber laser technology hinder the application in PAM, which requires the laser source to provide proper wavelength covering the molecular fingerprint, nanosecond pulse duration, and sufficient pulse energy (at least hundreds of nanojoules) for achieving efficient PA imaging. There are two most common methods of pulsed fiber laser generation in the MIR wavelength region, based on the current state of the art summarized in Table S1 in the Supplementary Material:^23^^–^^31^ (1) lasers based on rare-Earth-ion-doped fiber. Rare-Earth-ions-doped (${\mathrm{Er}}^{3+}$ and ${\mathrm{Dy}}^{3+}$) fluoride gain fibers can achieve lasing at wavelengths around 3  μm.^23^^–^^27^ Q-switching is a typical method for generating nanosecond laser pulses with high pulse energy.^23^^,^^26^^,^^27^ However, the wavelength tuning range is limited by the gain bandwidth of rare earth dopants: (2) supercontinuum (SC) fiber lasers. SC fiber lasers are known with a broad spectral coverage range from ultraviolet to far-infrared region.^28^^–^^31^ However, the output power spectral density is generally low ranging from tens of microwatts per nanometer to a few milliwatts per nanometer and is limited by the risk of damage to the end-face of the soft-glass fibers when using higher pump powers. Besides, although SC fiber lasers can cover a broad spectrum, they are not inherently wavelength-selective unless incorporated with additional components such as filters or tunable optics.^32^

In this work, we developed a novel MIR fiber laser technology for PA imaging for the first time. Specifically, the fiber laser emits high-energy nanosecond pulses at the absorption peak of myelin at 3.4  μm wavelength to image the neurons in the brain slice.^10^^,^^12^ The mechanism of the proposed laser is based on the stimulated Raman scattering (SRS) in gas-filled anti-resonant hollow-core fiber (ARHCF), which is an emerging fiber technology, that allows confinement of the laser beam within its hollow region resulting in very strong light-gas (atomic or molecular) interactions (thus efficient SRS frequency).^33^^,^^34^ This property not only enables the generation of strong pulses over a broad wavelength range from UV to MIR (as summarized in Table S2 in the Supplementary Material)^35^^–^^40^ but also is not limited to the damage threshold of the glass. Therefore, compared with other non-linear media, gases are considered, in a sense, self-healing, unlike solids which can become permanently damaged^34^ and thus allowing energy and intensity scaling of non-linear devices to their most extreme limit.^41^ Several studies have already demonstrated MIR laser pulse generation using gas-filled ARHCFs,^39^^,^^42^^–^^48^ and in this work, we developed a laser source platform with proper properties for PAM applications in biology settings and were able to employ it to achieve a PA imaging of neurons in mouse brain slices. This highlights its potential to broaden the spectral range beyond the existing fiber lasers utilized in PAM, such as standard doped silica fibers.^19^^–^^21^^,^^49^^–^^51^

## 2 Material and Methods

**Fig. 1:**
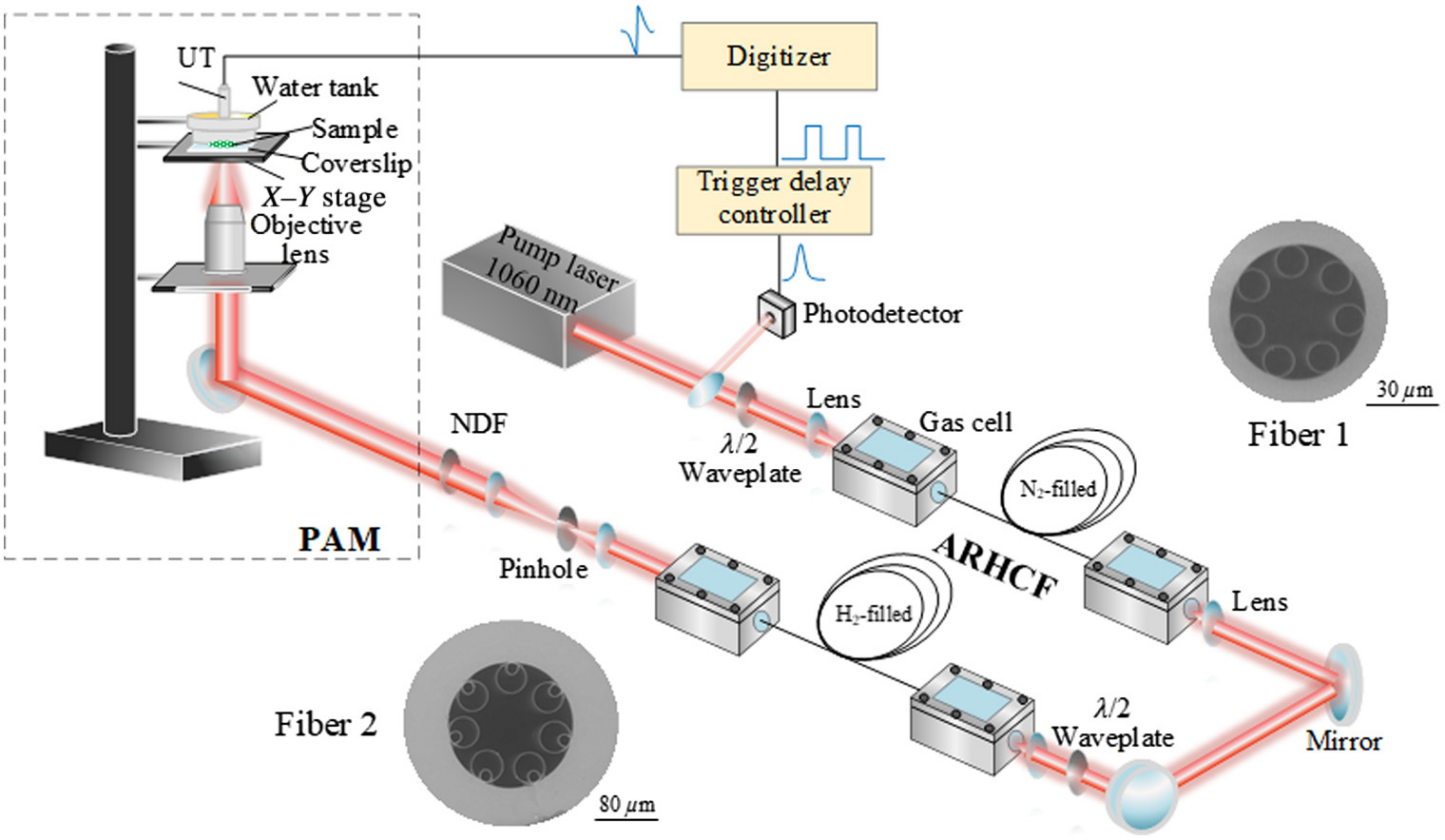
Overall MIR-PAM system. The system consists of a two-stage cascaded gas-filled ARHCF laser followed by a transmission mode PAM. Fibers 1 and 2: SEM images of the first- and second-stage ARHCFs, respectively. ARHCF, anti-resonant hollow-core fiber; NDF, neutral density filter; UT, ultrasound transducer; PAM, photoacoustic microscopy.

Figure 1 shows the configuration of the entire system combining the proposed MIR gas-filled fiber laser source and the PAM.

### 2.1 Cascaded Gas-Filled ARHCF Laser

The MIR gas-filled fiber Raman laser source at 3.4  μm is based on a configuration of cascading two gas-filled ARHCFs. To efficiently convert the conventional NIR to the MIR at the selected 3.4  μm wavelength, here, the second-stage ARHCF is filled with pure hydrogen ($H_{2}$), offering a long vibrational Raman stokes (VRS) coefficient of $4155\;{\mathrm{cm}}^{-1}$ and also a relatively high gain coefficient compared with other Raman active gases.^52^ This requires a pump wavelength of ∼1.4  μm, which is outside the gain range of the known rare-Earth-ion-based fibers. Therefore, we first generated the required ∼1.4-μm laser using the first-order VRS ($2331\;{\mathrm{cm}}^{-1}$) of $N_{2}$ in the first ARHCF stage,^53^ pumped by a Yb-doped fiber laser at the 1-μm region. The pump laser has an all-fiber structure consisting of a modulated diode laser seed followed by a Yb-doped fiber amplification module, emitting a pulse train with a repetition rate of 1.2 kHz, ∼3.7-ns pulse duration, energy of ∼98  μJ (measured by an energy meter, PE9-ES-C, Ophir Optronics, Jerusalem, Israel), and 0.128-nm linewidth at a 1060-nm wavelength.^32^ A λ/2 waveplate is placed at the output of the amplifier for adjusting the polarization orientation to acquire the highest Raman conversion efficiency in the subsequent gas-filled ARHCF system. Then, the beam is coupled into the $N_{2}$-filled ARHCF to generate the first-order VRS line at 1409 nm,^53^ which is then used as a pump for the second-stage ARHCF, to generate the Raman laser at MIR 3.4-μm wavelength.

The scanning electron microscopy (SEM) images of the two ARHCFs used in our experiments are shown in Fig. 1. The first-stage ARHCF (fiber 1 in Fig. 1) is a 16-m-long single-ring nodeless ARHCF, consisting of seven rings with a diameter of 16.1  μm and a wall thickness of ∼323  nm, forming a negative-curvature core shape with an inner jacket tube diameter of 32.8  μm. The simulated loss spectrum of the fiber is shown in Fig. S1(a) in the Supplementary Material, with a loss value of ∼0.02  dB/m at 1060 nm and ∼0.05  dB/m at 1409 nm.^53^ Based on the measured fiber’s parameters, the simulation was implemented based on the finite-element method using the COMSOL software with parameter settings in Ref. 54. A fine mesh size ranging from λ/6 to λ/4 was adopted to ensure the accuracy of our simulation. A perfectly matched layer was used outside the fiber structure as boundary conditions to accurately determine the leakage loss. The absorption coefficient of fused silica used in the simulation is from Ref. 55. The surface scattering loss was ignored because of the negligible surface roughness of the silica ARHCF compared with the wavelength in the IR region.^56^ The second-stage ARHCF (fiber 2 in Fig. 1) is 5 m long with a nested cladding structure and a core diameter of 82  μm.^32^ The diameter for external and internal capillaries are 40.3 and 13.6  μm, and the wall thickness are 987 nm and 1.37  μm, respectively. This ARHCF has a loss of only ∼0.004  dB/m at 3409 nm [see Fig. S1(b) in the Supplementary Material], which is a critical condition for the efficient generation of the 3.4-μm Raman laser. In addition, the nested structure of the second ARHCF significantly suppresses the bend loss and therefore allows a bending diameter of ∼40  cm facilitating the development of a compact PAM system. The first ARHCF can be also coiled with ∼40  cm diameter without high bending loss due to the small core diameter.^57^

### 2.2 Mid-Infrared Photoacoustic Microscopy

The generated 3.4-μm Raman laser beam is then expanded and coupled into the PAM. The output energy is properly attenuated using a neutral density filter (NDF, NDC-50C-2M, Thorlabs, Newton, New Jersey, United States) to avoid sample damage. The beam is focused by a reflected IR objective lens (PIKE, 40×, 0.78 NA) which has a designed obscuration of 42.9%. The PAM is performed in a transmission mode, and the sample is placed above a transparent sapphire-based coverslip (#18-471, Edmund Optics, Barrington, New Jersey, United States). Then, a water tank with a flat polymer bottom surface (μ-Dish, ibidi, Fitchburg, Wisconsin, United States) with 200  μm thickness is placed tightly above the sample to fix and flatten the sample surface and to couple the acoustic signal into the distilled water filled in the tank. The water tank and the sample are combined as an integrated part held by a high-resolution X−Y stage (8MTF-75LS05, Standa, Vilnius, Lithuania) driven by a stepper and direct current (DC) motor controller (8SMC5-USB, Standa) to enable the sample scanning with a minimum step size <10  nm and a maximum speed of 35,000 steps/s. A focused immersive ultrasound transducer (UT, Precision Acoustic, Dorchester, United Kingdom) with a central frequency of 20 MHz is immersed within the distilled water in the tank. The UT has an acoustic focal length of 8 mm and a diameter of 10 mm and is coaxially aligned with the reflective IR objective lens. The detected PA signals are filtered by two analog filters (mini-circuits, 1-MHz long-pass filter, and 27-MHz low-pass filter) then amplified by a low-noise wideband amplifier (Spectrum Instrumentation, Großhansdorf, Germany) and finally received with a high-speed digitizer (M4i.4421-x8, Spectrum Instrumentation) for data processing. The digitizer, integrated into a computer, operates at 250  MS/s sampling rate with a voltage resolution of 16 bits.

An external trigger delay controller (AeroDiode) synchronizes the scanning and data acquisition. A small fraction of the pump pulse energy is extracted from the pump beam and is recorded by a NIR photodetector (DET08C/M, Thorlabs) as a trigger signal, as shown in Fig. 1. Then, the output signal from the photodetector is connected to the input side of a trigger delay controller as the input trigger signal. Once the trigger delay controller detects the pump pulse signal, a square signal is generated and acts as the PA trigger for X−Y stage movement and signal synchronization. The PA signal is recorded ∼5  μs after the laser pulse and ∼0.2  μs after the square trigger signal at a repetition rate of 1.2 kHz. During the sample scanning, to minimize noises and enhance the signal-to-noise ratio, 100 pulses (A-lines) are averaged corresponding to a ∼83-ms dwell time.

### 2.3 Brain Sample Preparation

To investigate the performance of our MIR-PAM in real brain tissue, wild-type adult mice were employed. The procedure to prepare the brain slices presented below is approved by the Animal Experiments Inspectorate under the Danish Ministry of Food, Agriculture, and Fisheries, and all procedures adhere to the European guidelines for the care and use of laboratory animals, EU Directive 2010/63/EU. A Long–Evans wild-type adult rat was anesthetized by intraperitoneal injection of 200  mg/kg sodium pentobarbital. Hereafter, the level of anesthesia was assessed by pedal reflex pain response to firm toe pinch. Once a sufficient level of anesthesia was reached, the chest cavity was opened to expose the heart. A catheter, connected to a peristaltic pump (minipuls3, Gilsion, Middleton, Wisconsin, United States), was inserted into the left ventricle of the heart, and a small incision was made into the vena cava inferior for perfusion. The perfusion of the rat was performed with 30 ml of 1X phosphate-buffered saline (PBS) followed by 30 ml of 4% paraformaldehyde (PFA) at the rate of $7.8\;\mathrm{ml}\;\min^{-1}$. The rat was subsequently decapitated using a guillotine. The brain was dissected out and directly put into ice-cold 4% PFA. After fixation for 4 h at 4°C, the brain was transferred to 30% sucrose (w/v) for cryoprotection at 4°C. A vibratome (VT1200, LEICA, Wetzlar, Germany) was then used to section the brain into slices. To facilitate sectioning, the brain was embedded in 3% agarose gels prepared with a method similar to the one for preparing the 0.6% gels described above. The slicing was conducted in ice-cold 1X PBS. A similar process has been also followed in Ref. 58. The brain slice has a thickness of 400  μm. During the PAM imaging, the slice is kept in 1X PBS at room temperature.

## 3 Results and Discussion

### 3.1 Gas-Filled Hollow-Core Fiber Laser Source

**Fig. 2:**
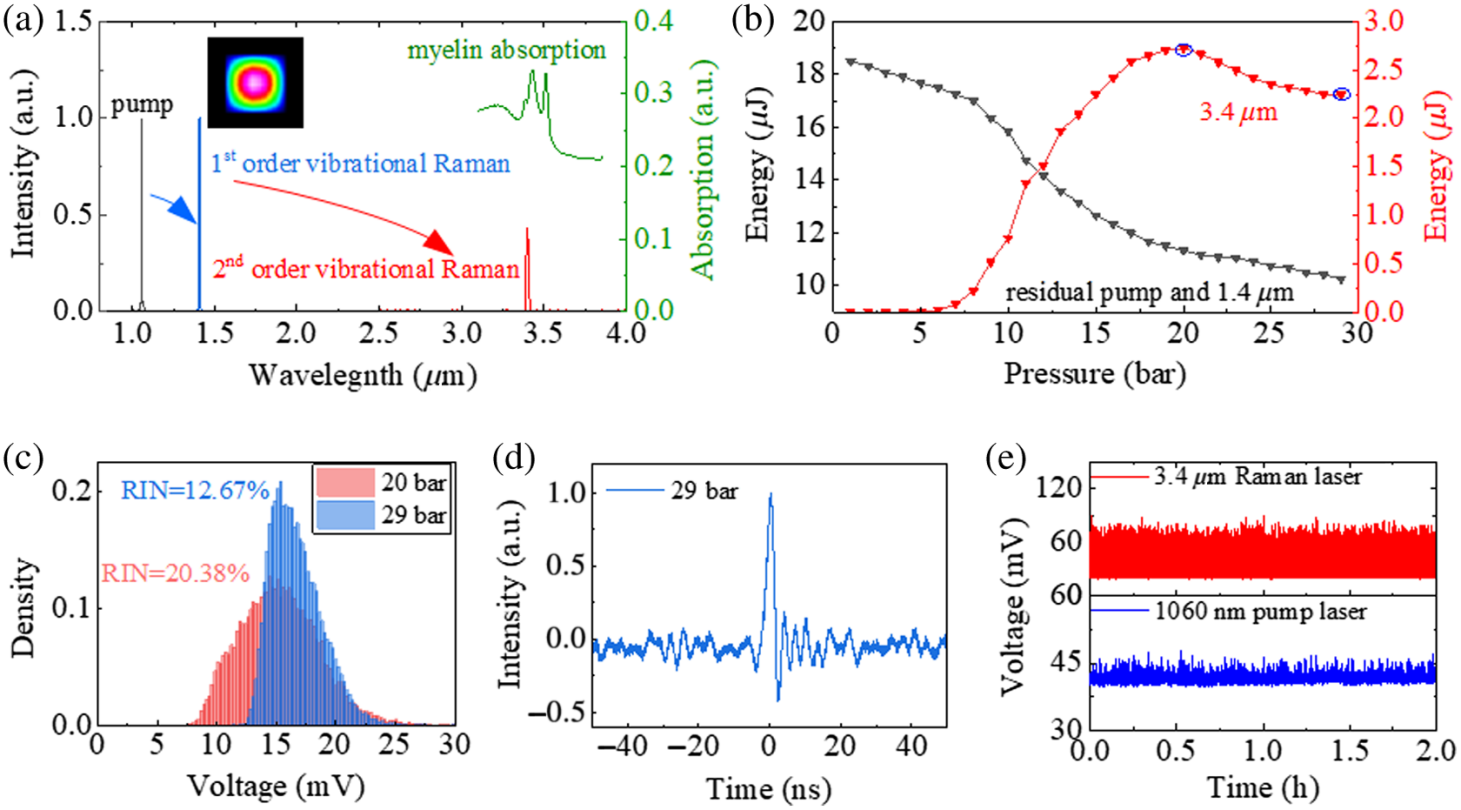
Characterization of the gas-filled ARHCF laser source. (a) Measured spectra including the pump line and Raman lines generated from the cascaded ARHCFs. The right axis shows the absorption spectrum of myelin extracted from.^10^ Inset: beam profile of first Raman pulse at 1409 nm. (b) Pulse energy evolution of the 3.4  μm Raman pulse as a function of $H_{2}$ pressure. (c) Histograms of the pulse peak intensity of the 3.4  μm Raman pulse at 20 and 29 bar, respectively. (d) Pulse profile of the 3.4-μm Raman laser. (e) Pulse peak intensity monitoring of the 1060-nm pump and the 3.4-μm Raman lasers over 2 h.

Figure 2(a) presents the spectra of the pump laser as well as Raman Stokes lines, measured using an infrared spectrometer (Spectro320 Instrument Systems) with a resolution of 0.14 nm. The Gaussian-like beam profile of the first Raman pulse, measured by a beam profiler (BP109-IR2, Thorlabs), indicates that the Raman laser operates in the fundamental mode. To maximize the output pulse energy, first, we measured the pulse energy of the 1409-nm Raman line output of the first-stage ARHCF in terms of the $N_{2}$ pressure.^53^ The Raman line appears at 9 bar pressure, and the pulse energy reaches its maximum of up to ∼26.5  μJ at ∼15-bar pressure, corresponding to a quantum efficiency of 45%.

By pumping the 1409-nm Raman line into the second-stage ARHCF, a Raman line at 3.4-μm wavelength is observed when the pressure is >5 bar, as seen in Fig. 2(a), with a linewidth of ∼1  nm. The average power of the 3.4-μm Raman laser was measured by extracting it from the residual 1.4-μm pump using a 2400-nm long-pass filter (FELH2400, Thorlabs, transmission 98% at 3.4  μm). Figure 2(b) shows the pulse energy evolution of the 3.4-μm Raman laser, as well as the total energy including both the Raman laser and residual pump at 1409 nm without using a filter, as a function of the $H_{2}$ pressure. The Raman pulse energy begins to rise at ∼5 bar, with the highest value being 2.75  μJ at ∼20  bar. As the pressure further increases, the energy starts to decrease slightly, because the Raman laser reaches its maximal pulse energy at a shorter fiber length. The long-term stability and noise performance of the Raman laser source were also characterized to provide a reference for implementing the subsequent PAM application. The noise performance of the MIR Raman pulses was measured in terms of the relative intensity noise (RIN) of pulse peak intensity at 20 and 29 bar, using a photodetector (100-MHz bandwidth, PDAVJ8, Thorlabs) connected to an oscilloscope (6-GHz bandwidth, MSO64B, Tektronix, Beaverton, Oregon, United States). Figure 2(c) shows the distribution of the measured histograms. At 20-bar pressure, the distribution has a Gaussian-like profile with a RIN of 20.38%. Compared with 20 bar, at 29-bar pressure, although the pulse energy slightly decreases to 2.25  μJ, a lower RIN of 12.67% is obtained. This is because the SRS process becomes more efficient toward higher $H_{2}$ pressure due to the further suppression of the transient SRS regime.^59^ Given this property, here, we set the pressure of $H_{2}$ to 29 bar, to better mitigate the acoustic signal fluctuation in the PAM application. Figure 2(d) shows a typical pulse profile of the 3.4-μm Raman laser. The pulse width is ∼2  ns, but the precision of this measurement is compromised because of the limited bandwidth of the MIR photodetector. This short nanosecond pulse gives a high peak power of ∼1.38  kW. This Raman laser also shows good long-term stability, as indicated by the 2-h monitoring results of the laser peak intensities of both the 3.4-μm Raman laser and its pump at 1060 nm. The average peak intensities and corresponding standard deviation of 2-min-long time bins over this time period were also calculated to better visualize the stability performance of the source and is presented in Fig. S2 in the Supplementary Material. The wavelength range of the MIR Raman laser can be tuned within a 10-nm range by thermally tuning the laser diode seed of the pump laser in the temperature range of 20°C to 35°C. The central wavelength of the pump source can be tuned from 1060 to 1061 nm, recorded from the spectrum by an infrared spectrometer (Spectro320 Instrument Systems) with a resolution of 0.14 nm, as shown in Fig. S3 in the Supplementary Material. The central wavelength of the generated first- and second-stage Raman lines is calculated based on the Raman shift coefficient of $N_{2}$ and $H_{2}$, respectively. As a result, it provides a tunable central wavelength range of ∼2  nm for the Raman line in the first stage and ∼10  nm for the MIR Raman line in the second stage from ∼3.391 to ∼3.401  μm. Employing a pump laser source with a larger wavelength tuning range will further expand the wavelength tunability.

### 3.2 Characterization of the MIR-PAM

**Fig. 3:**
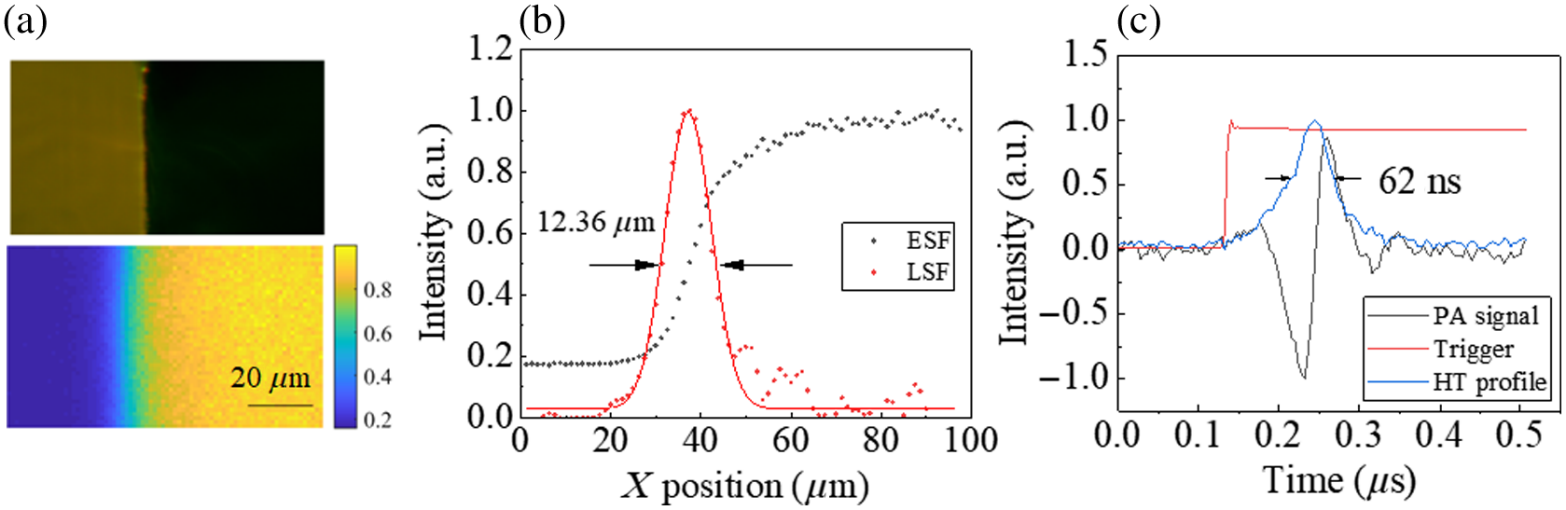
Resolution characterization of the MIR-PAM. (a) Optical and photoacoustic images of a sharp blade edge. (b) Fitted ESF and LSF extracted from PA image in panel (a). (c) Single-PA signal and its Hilbert transformation (HT) profile.

The lateral resolution of the PAM was first evaluated by imaging a sharp knife edge with a thickness of 100  μm. Figure 3(a) shows the optical image (top) and the corresponding PAM image (bottom). The pulse energy before and after the objective lens are ∼1.9 and ∼1  μJ, respectively, due to the 42.9% obscuration. The PAM images are obtained with a step size of 1.25  μm after averaging 100 pulses. The edge spread function (ESF) is acquired from the averaged raw data of the intensity, and the line spread function (LSF) is then calculated and fitted from the differential of the ESF, as shown in Fig. 3(b). From the full width at half maximum (FWHM) of LSF, the lateral resolution of our MIR-PAM is estimated to be ∼12.36  μm.

The axial resolution $R_{a}$ is estimated experimentally by the pulse width of the impulse response of a single PA signal. Figure 3(c) shows the PA signal and its envelope after Hilbert transformation (HT). The axial resolution is extracted from the FWHM of the envelope, which is 62 ns, corresponding to 95.5  μm in distance, given the propagation velocity of sound in the sample of c=1540  m/s.^60^ The axial resolution can also be numerically calculated by the velocity of the acoustic signal and the central frequency of the ultrasound transducer as $R_{a}=0.88c/B$, where B is the central frequency of the UT. In our case, the theoretical axial resolution is ∼67.8  μm.

### 3.3 *Ex Vivo* PAM

**Fig. 4:**
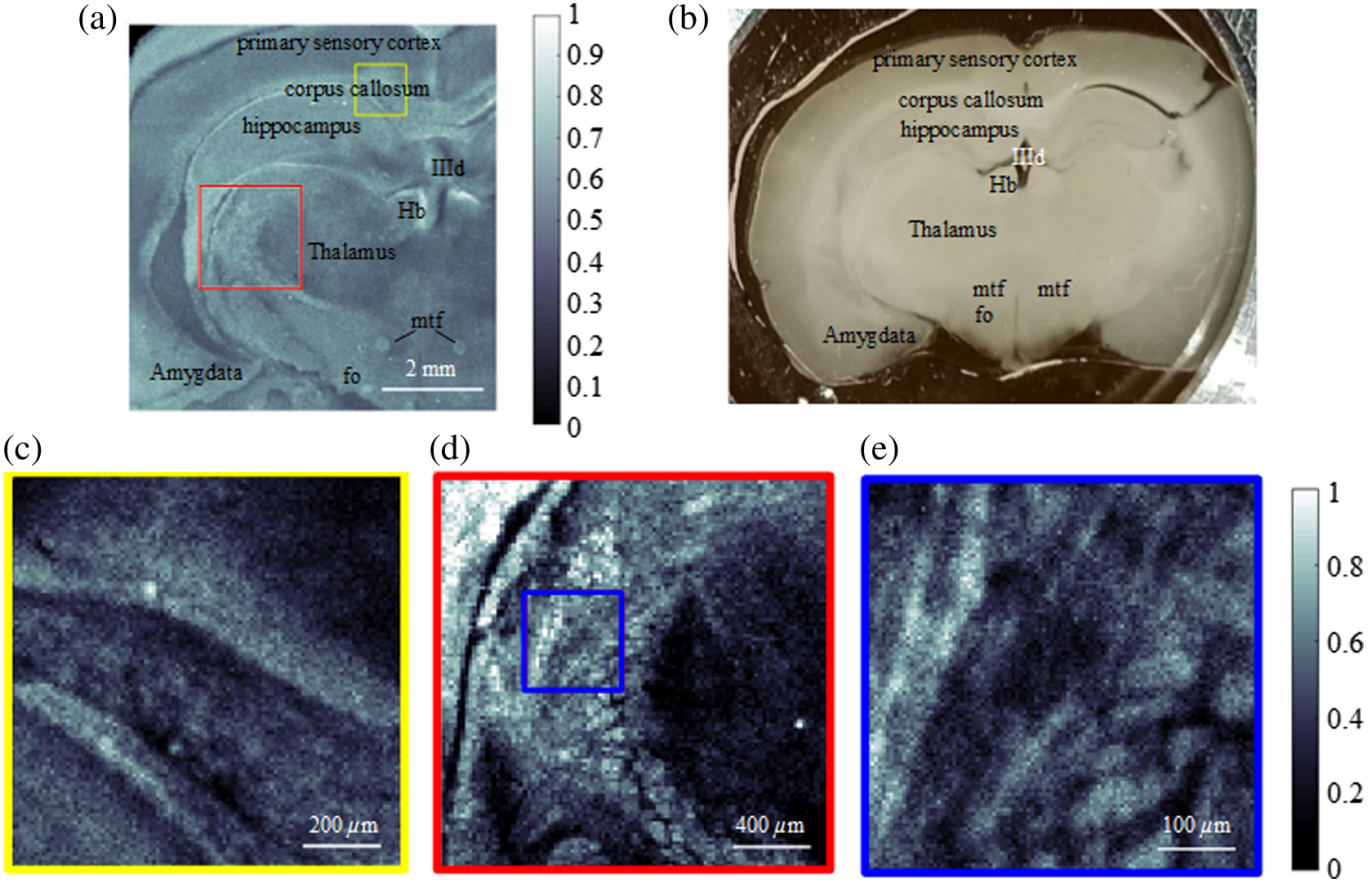
Image of the mouse brain. (a) *Ex vivo* PA image of the mouse brain slice. Hb, habenular nuclei; IIId, third ventricle; mtf, mammillothalamic fasciculus; fo, fornix. (b) Optical image of the mouse brain slice. (c) Enlarged view of the structure in the yellow box in panel (a) with a scanning step size of 10  μm. (d) Enlarged view of the structure in the red box in panel (a) with a scanning step size of 20  μm. (e) Enlarged view of the structure in the blue box in panel (d) with a scanning step size of 5  μm.

Figure 4(a) shows the *ex vivo* PAM image of the mouse brain slice with a thickness of 400  μm obtained by using the proposed 3.4-μm laser as a light source. The scanning step size is 40  μm, and the total scanning time is ∼40  min. HT was used for extracting the absolute amplitude of the acoustic signal, and then the image is post-processed by gamma transformation and a contrast enhancement of 0.5% saturation using the ImageJ software. In comparison with the optical image in Fig. 4(b), we can clearly see the outlines of the different brain regions. The myelin-rich regions, such as corpus callosum, are brighter than other regions. Small structures such as mammillothalamic fasciculus (mtf) and fornix (fo) can also be distinguished, exhibiting as bright spots, because they consist of heavily myelinated fibers.^10^^,^^61^^,^^62^ These characteristics are invisible in the optical image shown in Fig. 4(b). The regions with less myelin, such as the hippocampus, which mainly consists of gray matter, appear to be darker. Finally, due to the lack of myelin, the cavity structure (lateral ventricle IIId) looks like a void with clear boundaries.

Enlarged views of the corpus callosum and thalamus regions were scanned with a step size of 10 and 20  μm, respectively, as shown in Figs. 4(c) and 4(d). The border between the corpus callosum and the hippocampus is clearly visible in Fig. 4(c). In Fig. 4(d), patterns with a fish scale–like appearance are observable, which agree with other brain images reported in the literature.^10^ A further enlargement of the thalamus region, performed with a scanning step of 5  μm, is shown in Fig. 4(e). Here, we can observe some thick nerve fiber bundles that have been also verified and observed by other MIR-PAMs,^10^^,^^13^ demonstrating the ability of our new MIR-PAM Raman fiber laser for lipid-rich myelin imaging. All enlarged images required a scanning time of ∼20  min.

**Fig. 5:**
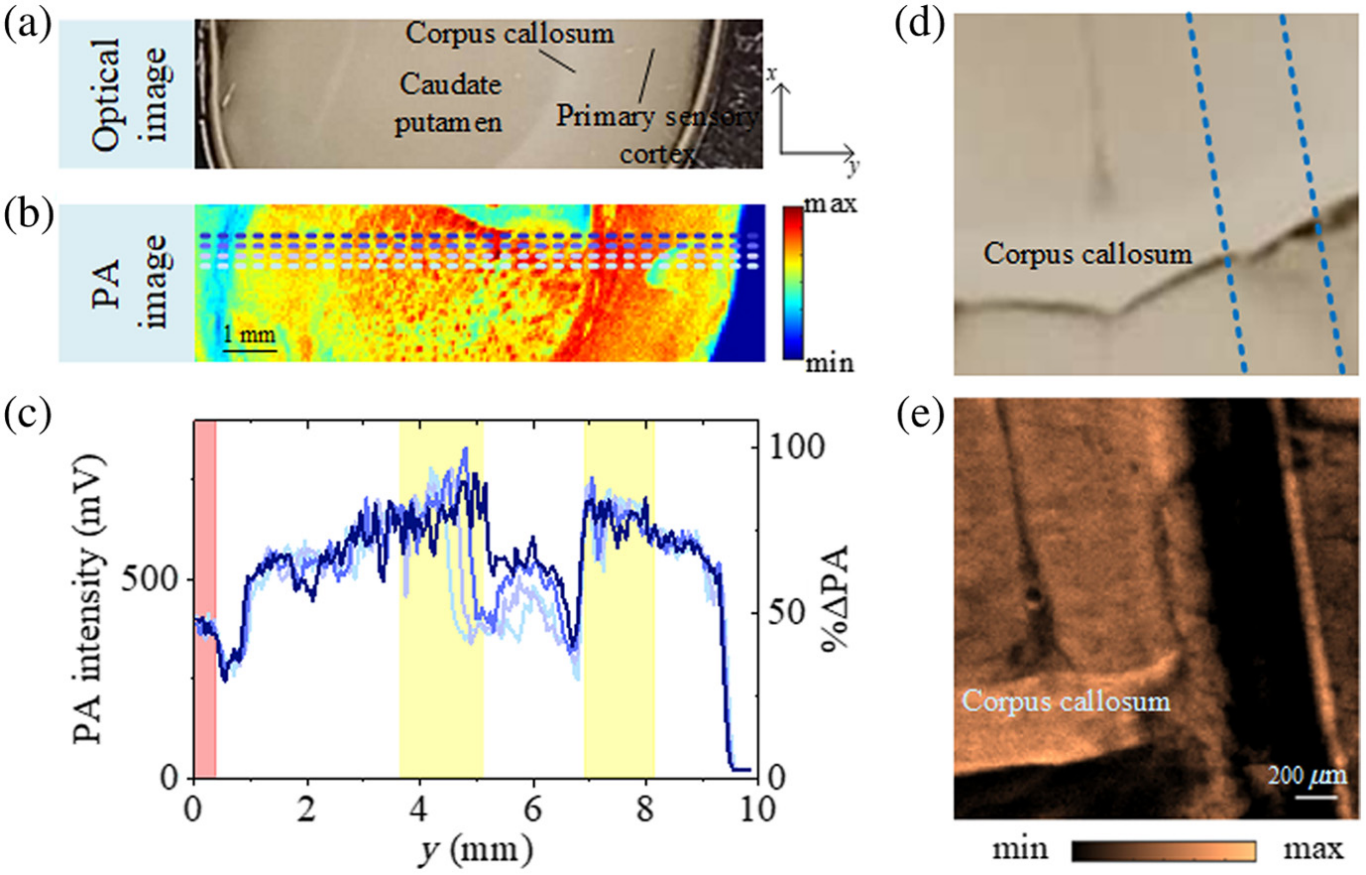
Image of the mouse brain. (a) Optical and (b) PA images of a 600-μm-thick mouse brain slice. The PA image is scanned with a step size of 50  μm. (c) PA signal intensity profiles along the dashed lines in panel (b) showing the contrast level in the water background (the pink-shaded region) and brain sample (the yellow-shaded regions) and calculated signal amplitude ratio to the maximum PA signal amplitude. (d) Optical and (e) PA images of a 600-μm-thick mouse brain slice implanted with a fiber. The PA image is scanned with a step size of 10  μm.

To examine the imaging contrast and penetration depth of the system, another brain slice sample with a larger thickness of 600  μm in the mouse brain was imaged with a scanning step size of 50  μm. Once again, the comparison between Figs. 5(a) and 5(b) shows that our system is able to highlight the higher density of myelinated neurons in regions such as the primary sensory cortex, corpus callosum, and caudate putamen. As shown in Fig. 5(c), the imaging contrast performance was evaluated from the unprocessed PA image by comparing the maximum PA signal amplitude from the water background (the pink-shaded region) and the neuron-rich areas (the yellow-shaded regions). The ratio of the signal amplitude from the water background to the highest signal amplitude from the neuron-rich areas is around 50%. More specifically, the SNR and contrast-to-noise ratio (CNR) can be calculated to be around 39:1 and 20:1 by the same procedures in Ref. 11, i.e.

$$\mathrm{SNR}=\frac{\left|{\mathrm{PA}}_{s}\right|}{\left|{\mathrm{PA}}_{n}\right|},$$ (1)

$$\mathrm{CNR}=\frac{\left|{\mathrm{PA}}_{s}-{\mathrm{PA}}_{b}\right|}{{\mathrm{PA}}_{n}},$$ (2)

where ${\mathrm{PA}}_{s}$, ${\mathrm{PA}}_{b}$, and ${\mathrm{PA}}_{n}$ are the peak-to-peak values from sample, background, and noise, respectively. The calculated CNR in this work is in agreement with the previously reported works of phospholipid membrane mapping in Hala cells (22:1) and lower than that of differentiated adipocytes (220:1)^11^ and white adipose tissue (344:1).^12^ This fact can be partly attributed to the higher concentration of triglycerides in the latter two kinds of cells and tissues with respect to the myelin and cell membrane. The large values of CNR and SNR we achieved demonstrate a limited effect of water background on the PA signal generation. Meanwhile, the PA image of another 600-μm-thickness sample was obtained with a scanning step size of 10  μm in the myelin-rich corpus callosum region. Here, a 510-μm-diameter polylactic acid (PLA) fiber was preciously implanted in the brain slice, at a depth at which it was not visible in a standard optical image [Fig 5(d)]. The successful visualization of the fiber with our MIR-PAM system [Fig. 5(e)] demonstrates the potential capability of PAM to detect fiber-based interfaces in brain experiments. Although we have demonstrated the PA imaging of a thicker sample, the penetration depth should be evaluated due to the water attenuation in MIR. The maximum depth can be determined as the width of $1/e^{2}$ of a Gaussian curve fitted to the HT profile of the PA signals.^17^ This is because even imaging at depths where irradiation has dropped to less than 14% ($1/e^{2}$) of its initial value can generate a detectable PA signal. We showcased a single raw PA signal in the neuron-rich area from the 600-μm-thick brain sample and calculated the HT profile of the PA signal (as presented in Fig. S4 in the Supplementary Material). From the Gaussian fit profile, we obtained an estimated penetration depth of ∼209  μm. The evaluated penetration depth here is lower than the 575-μm penetration depth for the fat–polyamide–suture phantoms^17^ and higher than the 90  μm for the acinar glands^17^ at the same wavelength, a fact that can be attributed to different sample characteristics in terms of scattering and absorption.

## 4 Discussion and Conclusion

This paper aims to create a new avenue of the emerging gas-filled Raman laser technology for MIR-PAM. As a proof-of-concept, a high-energy Raman laser at ∼3.4  μm was developed and employed for brain imaging. This work marks a milestone for MIR-PAM that uses a compact fiber laser source, which are in an early stage due to the limitation in either gain bandwidth or pulse energy of the existing fiber laser sources. On the other hand, a gas-filled ARHCF laser achieves a high peak power compared with the commonly used QCLs which, although having the advantage of small footprints can generally only deliver a few watts of peak power pulses. Although the wavelength range of the laser source in this work is limited to a small tuning range compared with QCLs, this challenge could be addressed by incorporating a tunable pump laser or replacing the gas medium. For example, by expanding the pump wavelength range to 1015 to 1115 nm which is the typical Yb-doped fiber gain range, the wavelength of the second-stage MIR pulse can be extended to a larger range of 1058 nm.^32^^,^^63^ This work can be further extended to multispectral PAM by operating the gas-filled fiber laser with the reconfigurable multiple spectral lines spanning from UV to NIR region. By filling $H_{2}$ in the same fiber in the first stage, a multiwavelength laser source can be achieved from UV (down to ∼328  nm) to NIR (∼2200  nm).^63^ The dedicated selection of the wavelengths makes the Raman lines exactly overlap with the absorption spectra of different chemical chromophores to enable selective mapping of several absorbers. Furthermore, we anticipate that future studies could also demonstrate practical applications with similar systems in mapping plastic-based materials which have an absorption peak at ∼3.4  μm due to the similar carbon-to-carbon (C─C) bond structures or ${\mathrm{CH}}_{2}$ groups.^64^ Although this work still involves the free-spacing coupling to ARHCF, an all-fiber structure will be expected in our future work, given the recent progress on the low-loss splicing of ARHCFs.^42^^–^^44^

## Supplementary Material



## Data Availability

The data of this work are available from the corresponding author upon reasonable request.
