# Supplementary material for: Mid-infrared photoacoustic brain imaging enabled by cascaded gas-filled hollow-core fiber lasers
Source: Neurophotonics. 2024 Nov 26;11(4):045012. doi: 10.1117/1.NPh.11.4.045012 (PMC11589470; doi:10.1117/1.NPh.11.4.045012)
Supplement: Supplementary file 1 [file NPh_011_045012_SD001.pdf]

Supplementary Material

Mid-infrared photoacoustic brain imaging enabled by cascaded gas-filled hollow-core fiber lasers

Cuiling Zhang,[a] Kunyang Sui,[a,b] Marcello Meneghetti,[a,b] Jose Enrique Antonio-Lopez,[c] Manoj K. Dasa,[d] Rune W. Berg,[b] Rodrigo Amezcua-Correa,[c] Yazhou Wang,[a] and Christos Markos,[a,e]

[a]*DTU Electro, Technical University of Denmark, 2800 Kgs. Lyngby, Denmark*

[b]*Department of Neuroscience, University of Copenhagen, 2200 Copenhagen, Denmark*

[c]*CREOL, The College of Optics and Photonics, University of Central Florida, Orlando, Florida 32816, USA*

[d]*NKT Photonics A/S, Blokken 84, Birkerød DK-3460, Denmark*

[e]*NORBLIS ApS, Virumgade 35D, 2830 Virum, Denmark*

SUPPLEMENTARY NOTE 1. State-of-the-art of MIR fiber laser

Table.S1 Summary of representative MIR fiber lasers above 3-μm wavelength

Modulation system	Wavelength (μm)	Fiber material	Pulse duration (ns)	Pulse energy (μJ)	Year
Mode-locked	3.489	Er^{3+} doped fluoride gain fiber	34.6	0.0001	2018[23]
	3.400-3.612		0.053	0.0001	2019[24]
	2.97-3.30	Dy^{3+} doped fluoride gain fiber	0.033	0.0027	2018[25]
Q-switched	3.46	Er^{3+} doped fluoride gain fiber	2050	1.83	2018[23]
	3.4-3.7		1020	5.29	2020[26]
	2.97-3.23	Dy^{3+} doped fluoride gain fiber	270	12	2019[27]
Nonlinear super-continuum	1-3.2	Silica-ZBLAN fibers	2	260	2007 [28]
	1.6-11	Silica-ZBLAN-As_2S_3-As_2Se_3 fibers	1.1	0.173	2018[29]
	2-6.5	Butt coupled ZBLAN-chalcogenide fibers	0.1	1.88	2021[30]
	0.2-4	ARHCF	0.0001	5	2019[31]

Note: ZBLAN: ZrF_4-BaF_2-LaF_3-AlF_3-NaF. ARHCF: anti-resonant hollow-core fiber.

Table.S2 State-of-the-art of MIR gas-filled ARHCF lasers

Year	Gas	Wavelength (μm)	Pulse duration (ns)	Pulse energy (μJ)
2018[42]	CH_4	2.8	~0.4	14
2017[43]	C_2H_2	3.11, 3.17	~1	1.4
2018[44]	CH_4	2.8	0.012	113
2019[45]	NO_2	4.6	/	0.08
2017[46]	H_2	4.42	~2	10
2018[47]	H_2/D_2	2.9, 3.3, 3.5	3.4	1.3
2019[48]	H_2	4.4	~2	1200
2020[49]	H_2	4.2	~4.5	17.6
This work	$N_2(1^{st})/H_2(2^{nd})$	3.4	~2	2.75

SUPPLEMENTARY NOTE 2. Loss spectrum of ARHCFs

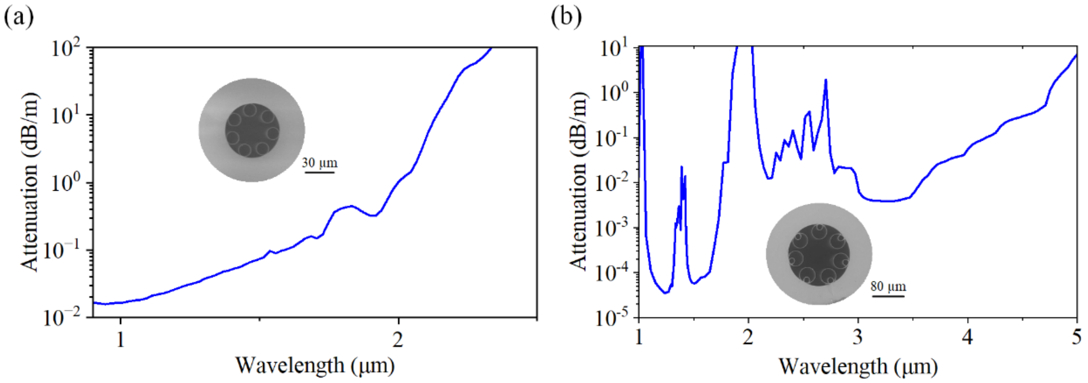

Fig. S1. Simulated attenuation spectra of **(a)** the first stage ARHCF and **(b)** the second stage ARHCF.

For the first stage ARHCF, the simulated attenuation coefficient is ~0.02 dB/m at 1060 nm and ~0.05 dB/m at 1409 nm, respectively. For the second stage ARHCF, the attenuation coefficient is ~0.005 dB/m at 1409 nm and ~0.004 dB/m at 3.4 μm, respectively.

SUPPLEMENTARY NOTE 3. Mean with STD within 2 minutes segment over the course of 2 hours.

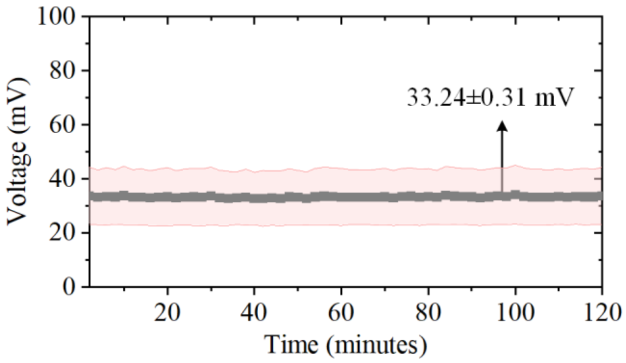

Fig. S2 The average peak intensities and corresponding standard deviation (STD) within a 2-minute-long time bins over the course of 2 hours monitoring in Fig. 2(e).

The pulse peak intensities of 3.4 μm Raman laser in Fig. 2(e) was monitored over ~2 hours by recording pulses with 60 ms average time separation between two adjacent pulses. The average peak intensities and corresponding standard deviation (STD) within each 2-minute-long time bin were calculated and presented in Fig. S2. The peak intensity of pulses fluctuates over a short time, but the averaged peak intensity remains stable over a long time, with a standard deviation of ~0.31 mV.

SUPPLEMENTARY NOTE 4. Tunable central wavelength range of pump laser, 1st Raman line, and 2nd Raman line.

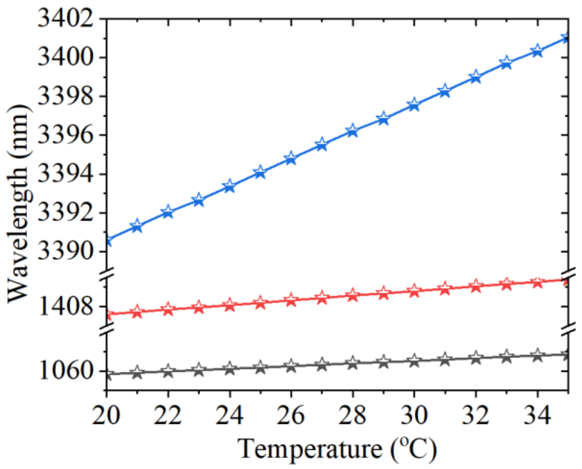

Fig. S3. The central wavelength of pump laser and Raman lasers at different temperatures. Black curve: the measured central wavelength of pump laser using an infrared spectrometer (Spectro320 Instrument Systems) with a resolution of 0.14 nm. Red curve: the calculated central wavelength of the 1st-stage Raman laser based on the Raman shift coefficient of nitrogen (N_2) and the measured pump wavelength [37]. Blue curve: the calculated central wavelength of the 2nd-stage MIR Raman laser based on the Raman shift coefficient of hydrogen (H_2) and the central wavelength of the 1st-stage Raman laser [37].

SUPPLEMENTARY NOTE 5. Evaluation of penetration depth

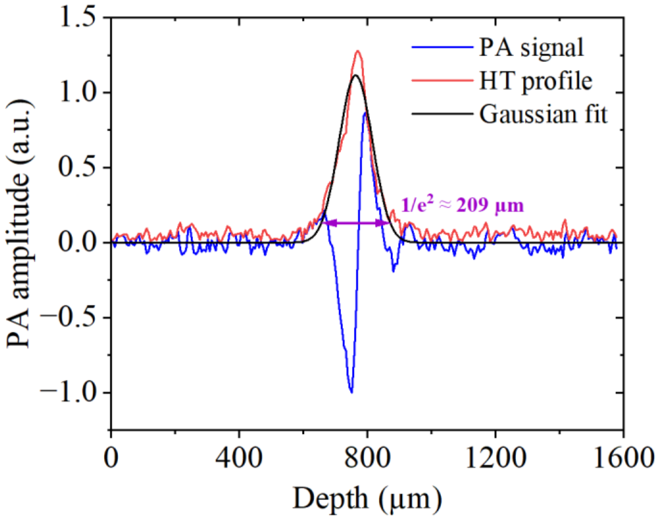

Fig. S4. A single raw PA signal and its Hilbert transformation (HT) profile to evaluate the penetration depth of the imaging system.
